# Anticoagulation for Stroke Prevention of Concomitant Atrial Fibrillation and End-Stage Renal Disease: Insights of Cardiologists and Nephrologists From India

**DOI:** 10.7759/cureus.32788

**Published:** 2022-12-21

**Authors:** Swetha ES, Santosh Taur, Namrata Kulkarni

**Affiliations:** 1 Internal Medicine, Pfizer India, Mumbai, IND; 2 Vaccines, Pfizer India, Mumbai, IND

**Keywords:** warfarin, apixaban, major bleeding, stroke prevention, atrial fibrillation, end-stage renal disease, chronic kidney disease

## Abstract

Introduction

Patients with concomitant atrial fibrillation (AF) and end-stage renal disease (ESRD) are at increased risk of thrombosis and bleeding. Diligent anticoagulant therapy that prevents major bleeding is essential for stroke prevention. There is a dearth of evidence and guidance on anticoagulation in this patient subset.

Methods

A validated questionnaire was sent to 500 physicians across India. Anonymized responses from 353 consenting physicians (275 cardiologists and 78 nephrologists) were analyzed.

Results

Most physicians opined that the risk of progression of chronic kidney disease (CKD) stages 2-4 to ESRD was 1-5%, and that >10% of patients with ESRD had concomitant AF. Most physicians perceived that the risk of ischemic stroke, major bleeding, and mortality was 30-40%, <15%, and >40% respectively in patients with concomitant AF and ESRD. The first critical goal for the management of these patients was ‘reduction of thrombotic risk’, followed by ‘prevention of bleeding’ and finally ‘prevention of ESRD progression’ (72.0%, 68.0%, and 67.1% participants, respectively). Most participating physicians (93.8%) preferred non-vitamin K antagonist oral anticoagulants (NOACs) over warfarin for stroke prevention, and most of the participating physicians (94.9%) preferred an adjusted dose rather than the standard dose of the NOAC. Most of the responses were similar between cardiologists and nephrologists.

Conclusion

According to the survey response, patients with concomitant AF and ESRD have an increased risk of thrombosis, bleeding, and mortality. NOACs with dose adjustment are the preferred modality for stroke prevention among cardiologists and nephrologists in India, with the primary goal of preventing thrombotic events.

## Introduction

The risk of developing atrial fibrillation (AF) is two to three times higher among patients with chronic kidney disease (CKD) compared to the general population [1]. The prevalence of AF is approximately 11 times higher among patients with end-stage renal disease (ESRD) compared to the general population [2]. The risk of thromboembolism due to AF is independently higher among patients with CKD, compared to those without CKD [3]. The annual incidence of stroke is five-fold higher in ESRD even in patients with CHA2DS2-VASc score of 0 [4]. The risk for stroke increases based on the CKD stage, and patients with ESRD undergoing hemodialysis (HD) are at an increased risk of developing AF [5]. According to the United States renal data system (USRDS), patients with ESRD and AF had a 1.6-fold higher rate of stroke than those without AF [6]. Stroke in ESRD has a very poor prognosis, with one in three being a fatal event and the majority resulting in death within a year. AF is also known to increase the risk of progression of CKD to ESRD [7].

Furthermore, these patients are prone to increased bleeding risk, primarily due to factors such as uremia-induced platelet dysfunction, impaired interaction between platelets and vessel walls, and the use of heparin during HD sessions [4,8]. The estimated risk of major bleeding is at least twice among patients with renal impairment, and at least thrice among patients who require dialysis, compared to patients with normal renal function [4]. Therefore, the anticoagulant strategy must be diligently and intricately balanced. The situation is further complicated by the lack of clarity on the most appropriate mode of anticoagulation in this specific patient subset.

Among the two major groups of anticoagulants, the vitamin K antagonist (VKA) warfarin is still commonly used for stroke prophylaxis in this patient population [9]. However, warfarin use among patients with ESRD has been found to carry more risks than benefits. There have been no randomized controlled trials (RCTs), and the observational data are also inconsistent. A meta-analysis of observational data suggested that warfarin significantly increased the risk of bleeding, without the benefit of reducing the risk of stroke [10]. Warfarin use in these patients has been associated with 10-times increased bleeding risk and a two-fold increased risk of hemorrhagic stroke [11,12]. Warfarin may also increase the risk of ischemic stroke in this population: this has been linked to microvascular calcification and calciphylaxis that sets in during ESRD, and this can further accelerate the decline in renal function [13]. Chronic renal failure may alter the intestinal, renal, and hepatic metabolism and transport of drugs, produce a clinically significant impact on drug disposition, and exaggerate various drug interactions. Other disadvantages of warfarin include a narrow therapeutic index and large interpatient variability [14].

Of the four non-VKA oral anticoagulants (NOACs) approved for stroke prevention in AF, apixaban depends the least on renal elimination (27%) and therefore could be the most suitable NOAC for this patient population [15]. However, the evidence suggesting the efficacy and safety of NOAC use for stroke prevention in this patient population is limited with only one RCT that recruited ESRD patients with AF (the RENAL-AF study) but was stopped prematurely and therefore its results can only be considered exploratory; this study did indicate that apixaban may be an alternative to warfarin for stroke prevention in patients with concomitant AF with ESRD [16]. It is an ongoing debate of “anticoagulation versus no anticoagulation,” “VKA versus NOAC” for this indication, and the “appropriate dose of NOAC” to be used for anticoagulation in this setting [3,17].

As a result of these unanswered questions, the choice of anticoagulation for this subset of patients is not clear. A recently published physician survey from Canada revealed that although most physicians prefer warfarin for this indication, various factors impacted anticoagulant selection, such as CHADS2 score, previous history of ischemia and calciphylaxis, physician specialty, and patient risk profile [18]. However, due to the lack of credible RCTs, the evidence on anticoagulation in this patient population currently depends on observational studies [19]. In India, the anticoagulation strategy in this patient population is largely influenced by the decision of the nephrologist, although the primary treating physician is a cardiologist. The limited evidence, guidance, and experience in this domain prompted us to undertake this survey. Specific objectives of the survey were to understand the perception and challenges of cardiologists and nephrologists surrounding the burden of ESRD with AF, the patient profile, and the knowledge and practice of the clinicians in its management while awaiting results from RCTs.

## Materials and methods

This pan-India, questionnaire-based, cross-sectional, online survey was undertaken between April 2021 and September 2021. Cardiologists and nephrologists with at least 10 years of experience in treating ESRD patients with AF, with their primary practice in any region of India, were invited to participate in the study; physicians from other specializations or located outside India were excluded from the study. All physicians provided informed consent before their participation in the survey.

The questionnaire for the survey was drafted after examining all available evidence pertaining to anticoagulation among patients with ESRD and AF, and the construct validity and reliability of the questionnaire were checked through pilot testing in an internal advisory board meeting. The questionnaire started with a brief overview of the study and consent to participate; the initial questions captured the basic details of the responding physician, followed by the questions that catered to the objectives of the study. Seven questions in the questionnaire were single-best-response type, and one question consisted of physicians ranking their preferences; there were no open-ended questions. While physicians were not required to disclose their names but only mention their initials, all remaining questions were required to be answered. The final version of the questionnaire used for the survey is available in the appendix.

The entire survey was conducted multimodally using emails, SMS, and telephonic channels. Eligible cardiologists and nephrologists were contacted by email or telephone during which an initial briefing was provided. The questionnaire was delivered to respondents consenting to participate in the survey through an email, which contained the link to the questionnaire in Microsoft forms. All participating physicians were followed up with gentle reminders by email, SMS, and calls. The objective was to obtain responses from at least 300 physicians; this target sample size was derived keeping in mind the estimated prevalence of AF with ESRD, and an estimated physician population of 2500 across India who would treat patients with concomitant AF and ESRD.

All responses were kept anonymous to ensure confidentiality. Data was entered in Microsoft Excel and was analyzed during November 2021 using IBM SPSS ver. 22. Pearson Chi-square test was used to perform comparisons of responses between cardiologists and nephrologists, and a p-value <0.05 was considered statistically significant; for the remaining analyses, descriptive statistics was used. Since this was a survey about the experiences and preferences of physicians in their practice, and no human clinical data was collected, ethics committee approval was not deemed to be necessary.

## Results

Out of the initial 500 physicians contacted, 353 consenting physicians completed the survey (70.6% participation rate); 275 (77.9%) were cardiologists, and the remaining 78 (22.1%) were nephrologists. The participating physicians came from all regions of India: North (103, 36.8%), South (80, 26.8%), West (68, 24.3%), East (15, 5.4%), Central (12, 4.3%), and Northeast (2, 0.7%); information related to the location was not disclosed by 73 respondents. All participating physicians had at least 10 years of clinical practice experience and had experience in managing patients with CKD, ESRD, and AF. The entire questionnaire took an average of 04:15 minutes to complete.

AF in the setting of ESRD

Most participating physicians (283/353, 80.2%) observed that 1-5% of all AF patients with CKD stages 2-4 progressed to ESRD; there was no significant difference (p=0.243) in the response pattern to this question by cardiologists and nephrologists (Table 1). Most participating physicians (295/353, 83.6%) observed that more than 10% of ESRD patients had incidental or concomitant AF; there was no significant difference (p=0.365) in the response pattern to this question by cardiologists and nephrologists (Table 2).

**Table 1:** Physician perception of proportion of patients having atrial fibrillation and chronic kidney disease stages 3-4 who worsen to develop end-stage renal disease AF: atrial fibrillation; CKD: chronic kidney disease; ESRD: end-stage renal disease

Proportion of AF+CKD patients worsening to ESRD	Overall (N=353)	Cardiologists (N=275)	Nephrologists (N=78)	P value
1-5%	283 (80.2%)	215 (78.2%)	68 (87.2%)	0.243
5-10%	26 (7.4%)	24 (8.7%)	2 (2.6%)	
10-15%	18 (5.1%)	(5.5%)	3 (3.8%)	
>15%	26 (7.4%)	21 (7.6%)	5 (6.4%)	

**Table 2:** Physician perception of proportion of patients with concomitant atrial fibrillation and end-stage renal disease AF: atrial fibrillation; ESRD: end-stage renal disease

Proportion of patients with concomitant AF and ESRD	Overall (N=353)	Cardiologists (N=275)	Nephrologists (N=78)	P value
0-1%	3 (0.8%)	3 (1.1%)	0	0.365
1-5%	23 (6.5%)	20 (7.3%)	3 (3.8%)	
5-10%	32 (9.1%)	27 (9.8%)	5 (6.4%)	
>10%	295 (83.6%)	225 (81.8%)	70 (89.7%)	

Complications of concomitant AF and ESRD

**Figure 1:**
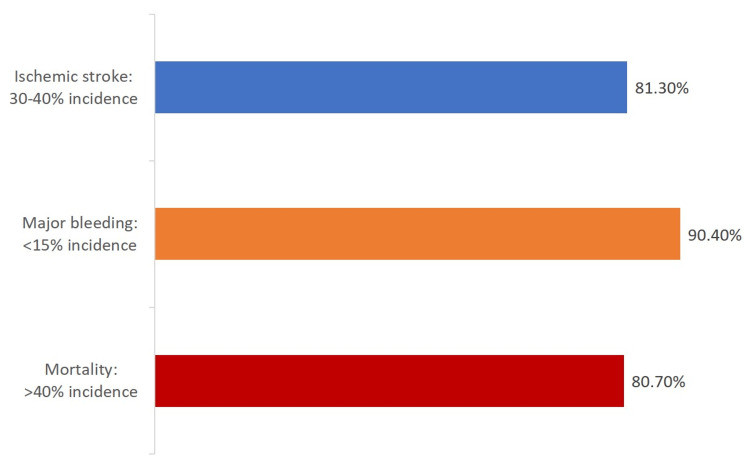
Physician perception of the incidence of three major complications among patients with concomitant atrial fibrillation and end-stage renal disease

Most participating physicians opined that the incidence of ischemic stroke among patients with concomitant AF and ESRD was 30-40% (287/353 responses, 81.3%), the incidence of major bleeding was <15% (319/353 responses, 90.4%), and the incidence of death was >40% (285/353 responses, 80.7%) (Figure 1, Table 3).

**Table 3:** Physician perception of incidence of complications among patients with concomitant atrial fibrillation and end-stage renal disease *statistically significant

Complication	Physician perception of incidence of complications	Overall (N=353)	Cardiologists (N=275)	Nephrologists (N=78)	P value
Ischemic stroke	<15%	25 (7.1%)	25 (9.1%)	0	0.025*
	15-30%	36 (10.2%)	28 (10.2%)	8 (10.3%)	
	30-40%	287 (81.3%)	217 (78.9%)	70 (89.7%)	
	>40%	5 (1.4%)	5 (1.8%)	0	
Major bleeding	<15%	319 (90.4%)	245 (89.1%)	74 (94.9%)	0.472
	15-30%	25 (7.1%)	22 (8.0%)	3 (3.8%)	
	30-40%	7 (2.0%)	6 (2.2%)	1 (1.3%)	
	>40%	2 (0.6%)	2 (0.7%)	0	
Mortality	<15%	24 (6.8%)	22 (8.0%)	2 (2.6%)	0.043*
	15-30%	30 (8.5%)	28 (10.2%)	2 (2.6%)	
	30-40%	14 (4.0%)	10 (3.6%)	4 (5.1%)	
	>40%	285 (80.7%)	215 (78.2%)	70 (89.7%)	

The response pattern between cardiologists and nephrologists was similar with respect to major bleeding. Although a significantly higher proportion of nephrologists than cardiologists felt that the incidence of ischemic stroke and mortality among patients with concomitant AF and ESRD was 30-40% (p=0.025) and >40% (p=0.043), respectively, the number of responding nephrologists was lower compared to the number of responding cardiologists for both options (Table 3).

Critical goal for management of patients with concomitant AF and ESRD

**Figure 2:**
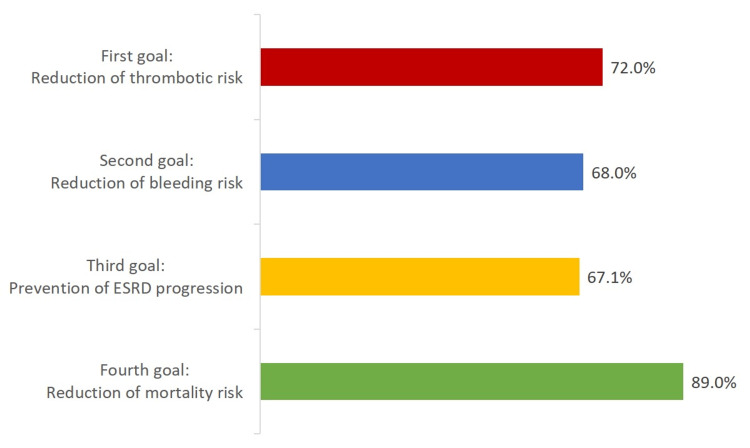
Physician perception of the critical goals for the management of patients with concomitant atrial fibrillation and end-stage renal disease ESRD: end-stage renal disease

According to most participating physicians, the first critical goal for the management of patients with concomitant AF and ESRD was a reduction of thrombotic risk (254/353 responses, 72.0%), followed by reduction of bleeding risk (240/353 responses, 68.0%), prevention of ESRD progression (237/353 responses, 67.1%), and reduction of mortality risk (314/353 responses, 89.0%) (Figure 2).

**Figure 3:**
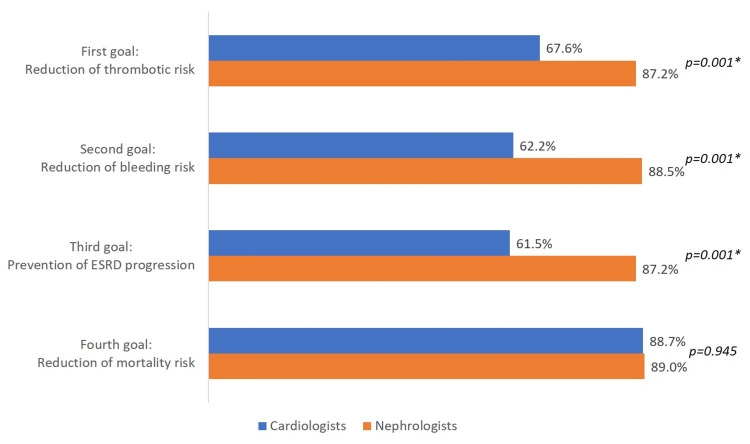
Differences in cardiologist and nephrologist perception of the critical goals for the management of patients with concomitant atrial fibrillation and end-stage renal disease *statistically significant; ESRD: end-stage renal disease

The ranking order of the preferences for treatment goals remained similar among cardiologists and nephrologists. Reduction of thrombotic risk was the first therapeutic goal for 67.6% of cardiologists and 87.2% of nephrologists; reduction of bleeding risk was the second therapeutic goal for 62.2% of cardiologists and 88.5% of nephrologists; prevention of ESRD progression was the third therapeutic goal for 61.5% cardiologists and 87.2% nephrologists; reduction of mortality risk was the fourth therapeutic goal for 88.7% cardiologists and 89.0% nephrologists (Figure 3, Table 4).

**Table 4:** Physician perception of incidence of complications among patients with concomitant atrial fibrillation and end-stage renal disease *statistically significant; ESRD: end-stage renal disease

Management Goal Ranking	Prevention of	Overall (N=353)	Cardiologists (N=275)	Nephrologists (N=78)	P value
First Goal	Thrombotic risk	254 (72.0%)	186 (67.6%)	68 (87.2%)	0.001*
	Bleeding risk	9 (2.5%)	7 (2.5%)	2 (2.6%)	
	ESRD progression	75 (21.2%)	71 (25.8%)	4 (5.1%)	
	Mortality	15 (4.2%)	11 (4.0%)	4(5.1%)	
Second Goal	Thrombotic risk	80 (22.7%)	75 (27.3%)	5 (6.4%)	<0.001*
	Bleeding risk	240 (68.0%)	171 (62.2%)	69 (88.5%)	
	ESRD progression	20 (5.7%)	18 (6.5%)	2 (2.6%)	
	Mortality	13 (3.7%)	11 (4.0%)	2 (2.6%)	
Third Goal	Thrombotic risk	16 (4.5%)	12 (4.4%)	4 (5.1%)	<0.001*
	Bleeding risk	89 (25.2%)	85 (30.9%)	4 (5.1%)	
	ESRD progression	237 (67.1%)	169 (61.5%)	68 (87.2%)	
	Mortality	11 (3.1%)	9 (3.3%)	2 (2.6%)	
Fourth Goal	Thrombotic risk	3 (0.8%)	2 (0.7%)	1 (1.3%)	0.945
	Bleeding risk	15 (4.2%)	12 (4.4%)	3 (3.8%)	
	ESRD progression	21 (5.9%)	17 (6.2%)	4 (5.1%)	
	Mortality	314 (89.0%)	244 (88.7%)	70 (89.0%)	

Drug preference for anticoagulation

**Figure 4:**
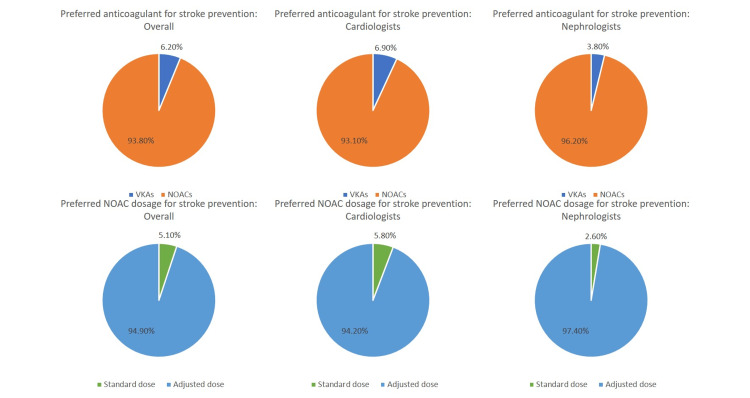
Physician preference of agent for anticoagulation and dose of NOAC for stroke prevention among patients with concomitant atrial fibrillation and end-stage renal disease VKA: vitamin K antagonists; NOAC: novel oral anticoagulants

A majority of participating physicians (331/353, 93.8%) preferred NOACs over VKAs for stroke prevention in patients with concomitant AF and ESRD, and most of the participating physicians (335/353, 94.9%) preferred to use a lower or adjusted dose rather than the standard dose of the NOAC for this indication. The response pattern to these questions was similar between cardiologists and nephrologists (Figure 4).

The pattern of responses to these two questions, namely, drug preference for stroke prevention and dose preference for NOACs in this indication, was not significantly different between physicians responding from North, South, and West India; responses from Central, East, and Northeast India were not included in this analysis due to the small sample size (Table 5).

**Table 5:** Region-wise analysis of physician preference of agent for anticoagulation for stroke prevention among patients with concomitant atrial fibrillation and end-stage renal disease VKA: vitamin K antagonists; NOAC: novel oral anticoagulants

	Preference	North (N=103)	South (N=80)	West (N=68)	P value
Preferred anticoagulant for stroke prevention	VKAs	2 (1.9%)	1 (1.2%)	4 (5.9%)	0.185
	NOACs	101 (98.1%)	79 (98.8%)	64 (94.1%)	
Preferred dosage of NOACs	Standard dose	4 (3.9%)	3 (3.8%)	1 (1.5%)	0.640
	Adjusted dose	99 (96.1%)	77(96.2%)	67 (98.5%)	

Data availability

All datasets leading to the conclusions of this research are available with the leading author upon reasonable request.

## Discussion

The prevalence of ESRD patients having incidental or concomitant AF is estimated at 27% among patients undergoing HD [5]. While similar data specific to the Indian setting is lacking, the physicians in our study opined that this number is greater than 10%, which agrees with the estimates. Around 1%-2% of CKD patients are known to progress to ESRD over time, which was similar to the opinion of the majority of the participating physicians in our survey [20].

A 2016 systematic review concluded that compared to ESRD patients without AF, the incidence of stroke and mortality are significantly higher among ESRD patients with AF (incidence of stroke: 1.9 vs 5.2 per 100 person-years, mortality: 13.4 vs 26.9 per 100 person-years, respectively) [2]. This was reflected in our study, in which most physicians opined that the incidence of ischemic stroke and mortality was higher among patients with concomitant AF and ESRD, in contrast to a relatively low risk of major bleeding. Stroke in ESRD has an extremely poor prognosis, with nearly 33% being a fatal event, and most patients succumb within a year [4]. In this context, concern about the fatal outcome of stroke after a thrombotic event in this patient subset could be the possible reason for most physicians in our study opting for the prevention of thrombotic risk as the most critical treatment goal.

With declining renal function, there is a gradual increase in the prevalence of AF and event rates of stroke and major bleeding, while there is a gradual decline in the available evidence for antithrombotic therapy to guide clinical decision-making [17]. In 2014, the US Food and Drug Administration (USFDA) additionally approved apixaban even for patients with creatinine clearance (CrCl) ≤ 15 ml/min without the need for dose reduction, unless the patients met any two of the three “ABC” criteria (age ≥ 80 years, body weight ≤ 60 kg, or serum creatinine ≥ 1.5 mg/dl); India is the second country to approve apixaban in patients with CrCl <15 ml/min for all approved indications [21]. Apixaban is approved for the prevention of stroke in AF, the treatment of venous thromboembolism (VTE), the prevention of secondary VTE, and the prophylaxis of VTE after total knee replacement (TKR)/total hip replacement (THR) surgeries. The American College of Cardiology/American Heart Association (ACC/AHA) in their 2019 guidelines have recommended that it might be reasonable to prescribe warfarin (INR 2.0-3.0) or apixaban for oral anticoagulation for patients with AF who have ESRD [22].

The other key factors that impact the selection of anticoagulants in patients with CKD and ESRD are deterioration of renal function and bleeding risk. Warfarin use accelerates the decline in renal function when compared to the NOACs [23]. Among the NOACs, there have been concerns about the use of rivaroxaban and dabigatran for stroke prophylaxis in patients with low renal reserves due to a high (84%) dependence of dabigatran on renal elimination, and a 70% increase in area under the curve (AUC) on plasma concentration with rivaroxaban in ESRD, which could lead to increased accumulation of these drugs, further worsening the bleeding risks [4,17]. An analysis of anticoagulant use through recent observational studies suggests that apixaban is gradually replacing warfarin over the years, despite the absence of strong guidelines that recommend apixaban over warfarin [23]. A recently published large real-world evidence study including Medicare beneficiaries suggested that apixaban, compared to warfarin, was not only associated with lower risks of CKD stage progression, but also with lower incidences of stroke, systemic embolism, and major bleeding when used for stroke prophylaxis in AF [23,24]. Perhaps due to these experiences, it appears that the physicians in India have also been adapting the use of NOACs, instead of warfarin, as also suggested by the responses in this survey, and this possibly reflects the challenges associated with warfarin [25].

In our survey, most physicians preferred to use a lower (or adjusted) dose of NOACs for stroke prevention. In the context of increased bleeding risk with declining renal function, this approach by the responding physicians implies their cautious approach towards this very high-risk group. A 2016 study by Wang et al. observed that administration of a single 5 mg dose of apixaban among patients with ESRD resulted in only a modest (36%) increase in apixaban exposure, and that hemodialysis did not have a significant impact on apixaban clearance with 14% removal [26]. Based on similar observations from another study, it was suggested that from a pharmacokinetic perspective, apixaban could be used without dose modification in these patients [27]. The data from Wang et al.’s study was used as the basis for apixaban dosing guidance in patients with ESRD by the USFDA [21,26]. Finally, the analysis of more than 25,000 beneficiaries with ESRD and AF in the Medicare claims database by Siontis et al. suggested that full-dose apixaban (5 mg twice daily) is associated with lower rates of stroke, systemic embolism, and death compared to reduced dose apixaban (2.5 mg twice daily) or warfarin [28]. However, the proportion of patients who required adjusted dose was higher in both RENAL-AF (Renal Hemodialysis Patients Allocated Apixaban Versus Warfarin in Atrial Fibrillation) trial and Siontis et al. study, which is 56% and 50%, respectively, in comparison to <5% in the ARISTOTLE (Apixaban for Reduction in Stroke and Other Thromboembolic Events in Atrial Fibrillation) study [16,28,29]. This was not unexpected, because the ARISTOTLE study excluded AF patients with CrCl <25 ml/min, and mandated a dose reduction if serum creatinine was >1.5 mg/dl and another criterion was also present concomitantly. However, the limited evidence appears to suggest that a full dose of apixaban (5 mg twice daily) should be preferred over a lower dose (2.5 mg twice daily) of apixaban for optimal outcomes in this patient subset unless the ABC criteria are met as mentioned on its label. These results should be interpreted with caution, and large-scale studies are warranted to establish the risk-benefit ratio of different NOACs in patients with AF on long-term dialysis.

Two recent physician surveys like ours have been reported in the recent past, and the overall findings are similar to the findings of our survey. In the survey by European physicians, there was a preference for warfarin, adjusted dose apixaban (2.5 mg twice daily), and lower dose edoxaban (30 mg once daily) over dabigatran and the Canadian physicians preferred warfarin for stroke prevention in patients with concomitant AF and ESRD [18,30].

Our study is not without limitations. The survey was conducted using purposeful sampling, which might have incorporated bias into the survey, and coupled with the low sample size, our study findings might not represent the opinion of the overall physician population in India. We cannot completely rule out respondent bias as well. Being a survey, the strength of the evidence generated is not high, but these results cannot be ignored, especially considering the dearth of RCT evidence in this domain. The association of the elderly population and ESRD in the setting of AF could not be analyzed, which might have substantiated the preference for an adjusted dose. Finally, there is a lack of uniform representation of all regions of India, and differences in rural-urban treatment preferences were not represented. However, our findings suggest that well-planned RCTs are required to explore the most appropriate treatment strategy for stroke prevention in ESRD patients with concomitant AF.

## Conclusions

According to the results of the physician survey presented here, patients with concomitant AF and ESRD have an increased risk of thrombosis, bleeding, and mortality. In our study, NOACs in reduced dose are the preferred modality for stroke prevention among cardiologists and nephrologists in India, with the primary goal of preventing thrombotic events. However, based on previous evidence, the risks of warfarin appear to outweigh the benefits when used in this patient population. The pharmacokinetics, safety, and efficacy profile of apixaban suggest that it may be the most suitable NOAC for stroke prevention in patients with concomitant AF and ESRD. Well-designed clinical trials that include this specific patient population are required to formulate appropriate management guidelines.

## Appendices

Questionnaire used for the survey: physicians' survey on patients with atrial fibrillation and end-stage renal disease

Patients with AF and ESRD are at increased risk of both stroke and major bleeding as compared to AF without ESRD. There is limited data and experience of using OACs in these patients. While awaiting the results from the ongoing randomized trials and recommendations, stroke prevention in patients with AF and ESRD/dialysis will remain challenging. This survey is to understand your experience of using OACs in patients with AF ESRD/dialysis.

This survey is to understand the perspectives and challenges of anticoagulation in patients with AF and ESRD/dialysis faced by cardiologists and nephrologists.

(All questions are compulsory)

1. Physician Details: Please enter your Initials, and Location: ___________

2 Date: _______

3 Please enter your Specialty

   a. Cardiologist

   b. Nephrologist

4. Physician Consent: We are conducting a survey of the Cardiologists and Nephrologists to understand clinical practices in the management of AF with ESRD. As a part of the survey, we will capture the responses provided by you in this document. We need your consent to proceed. Kindly confirm that you have understood the description of this survey, and your participation is voluntary. Pfizer may use the responses provided by you in any manner with the understanding that the same shall be used for study and analysis. You hereby consent to the use of this survey for future research purposes and publication related to this study.

   a. Yes

   b. No

5. What proportion of patients with AF and CKD stages 2-4 worsen to ESRD?

   a. 1-5%

   b. 5-10%

   c. 10-15%

   d. >15%

6. What proportion of patients with ESRD have incidental/concomitant AF?

   a. 0-1%

   b. 1-5%

   c. 5-10%

   d. >10%

7. What is the incidence of the following events in your patients with AF and ESRD?

   a. Ischemic Stroke: <15%/ 15-30%/ 30-40%/ >40%

   b. Major Bleeding: <15%/ 15-30%/ 30-40%/ >40%

   c. Mortality: <15%/ 15-30%/ 30-40%/ >40%

8. What is the critical goal of treatment in patients with AF and ESRD? Prevention of................ (Please select the following in the order of prioritization)

   a. ESRD progression

   b. Thrombotic risk

   c. Bleeding risk

   d. Mortality

9. Which OAC do you advise in patients with AF and ESRD for stroke prevention?

   a. VKA

   b. NOAC

10. What dose of OAC is generally used in patients with AF and ESRD?

   a. Standard

   b. Adjusted
